# Correction: Directed evolution of mevalonate kinase in *Escherichia coli* by random mutagenesis for improved lycopene

**DOI:** 10.1039/c9ra90021g

**Published:** 2019-03-18

**Authors:** Hailin Chen, Changqing Liu, Meijie Li, Haibo Zhang, Mo Xian, Huizhou Liu

**Affiliations:** CAS Key Laboratory of Bio-based Materials, Qingdao Institute of Bioenergy and Bioprocess Technology, Chinese Academy of Sciences No. 189 Songling Road Qingdao 266101 People’s Republic of China xianmo@qibebt.ac.cn zhanghb@qibebt.ac.cn; Sino-Danish College, University of Chinese Academy of Sciences No. 19 (A) Yuquan Road Beijing 100049 People’s Republic of China

## Abstract

Correction for ‘Directed evolution of mevalonate kinase in *Escherichia coli* by random mutagenesis for improved lycopene’ by Hailin Chen *et al.*, *RSC Adv.*, 2018, **8**, 15021–15028.

The authors wish to draw the readers’ attention to their closely related paper, published in *Microbial Cell Factories*,^1^ which should have been cited in this *RSC Advances* paper.

The authors regret that there is unattributed overlap in text between this *RSC Advances* paper and ref. 1. The authors confirm that new data has been reported in this *RSC Advances* article.

Two different rate-limiting enzymes in the lycopene synthetic pathway were studied using the same methods, mevalonate kinase (MK) in this paper and isopentenyl diphosphate isomerase (IDI) in ref. 1. In the *RSC Advances* paper, a directed evolution strategy was used to optimize the activity of MK to enhance the tolerance for farnesyldiphosphate (FPP) and geranylgeranyldiphosphate (GGPP), to enhance the affinity of mevalonate and MK, and to improve lycopene production. The catalytic mechanisms of both enzymes are very different; however improving their activities can improve lycopene production.

The Royal Society of Chemistry apologises for these errors and any consequent inconvenience to authors and readers.

## Supplementary Material
